# Effectiveness of the Comprehensive Approach to Rehabilitation (CARe) methodology: design of a cluster randomized controlled trial

**DOI:** 10.1186/s12888-015-0564-0

**Published:** 2015-07-22

**Authors:** Neis A. Bitter, Diana P. K. Roeg, Chijs van Nieuwenhuizen, Jaap van Weeghel

**Affiliations:** Tilburg University, Department of Social and Behavioural Sciences, Tranzo Scientific Centre for Care and Welfare, PO Box 90153, 5000 LE Tilburg, The Netherlands; GGzE Centre for Mental Health Care, PO BOX 909, 5600 AX Eindhoven, The Netherlands; Phrenos Centre of Expertise, PO Box 1203, 3500 BE Utrecht, The Netherlands; Parnassia Group, Dijk en Duin Mental Health Centre, PO Box 305, 1900 AH Castricum, The Netherlands

**Keywords:** Severe mental illness, Recovery, Recovery-oriented care, Rehabilitation, Strengths, CARe methodology

## Abstract

**Background:**

There is an increasing amount of evidence for the effectiveness of rehabilitation interventions for people with severe mental illness (SMI). In the Netherlands, a rehabilitation methodology that is well known and often applied is the Comprehensive Approach to Rehabilitation (CARe) methodology. The overall goal of the CARe methodology is to improve the client’s quality of life by supporting the client in realizing his/her goals and wishes, handling his/her vulnerability and improving the quality of his/her social environment. The methodology is strongly influenced by the concept of ‘personal recovery’ and the ‘strengths case management model’. No controlled effect studies have been conducted hitherto regarding the CARe methodology.

**Methods/design:**

This study is a two-armed cluster randomized controlled trial (RCT) that will be executed in teams from three organizations for sheltered and supported housing, which provide services to people with long-term severe mental illness. Teams in the intervention group will receive the multiple-day CARe methodology training from a specialized institute and start working according the CARe Methodology guideline. Teams in the control group will continue working in their usual way. Standardized questionnaires will be completed at baseline (T0), and 10 (T1) and 20 months (T2) post baseline. Primary outcomes are recovery, social functioning and quality of life. The model fidelity of the CARe methodology will be assessed at T1 and T2.

**Discussion:**

This study is the first controlled effect study on the CARe methodology and one of the few RCTs on a broad rehabilitation method or strength-based approach. This study is relevant because mental health care organizations have become increasingly interested in recovery and rehabilitation-oriented care.

**Trial registration:**

The trial registration number is ISRCTN77355880.

## Background

People with serious mental illnesses (SMI) experience numerous problems in their daily lives. Studies on employment, for instance, show that about 10–20 % of people with SMI have regular paid employment, 50 % work as volunteers or participate in organized day activities and approximately 40 % have no paid or unpaid employment at all [1, 2]. Furthermore, a lack of social contacts and loneliness is common among people with SMI [3–5]. Previous studies show that these people experience unmet needs in these areas, which results in a lower quality of life [6–9]. Hence, it is important that mental health care organizations address these needs and wishes. Psychiatric rehabilitation practices have been applied by mental health care organizations to increase social participation and improve quality of life over the last two decades [10, 11]. The goal of these practices is ‘to help individuals with complex, longer term mental health problems to develop the emotional, social and practical skills needed to live, learn and work in the community with the least amount of professional support’ [11–13]. Psychiatric rehabilitation is closely related to the concept of personal recovery. Personal recovery implies a client-oriented definition of recovery in which the emphasis lies more on personal development and growth than on symptom reduction. Important aspects of recovery are: hope, empowerment and the feeling of living a satisfying life despite symptoms of illness [14–22]. While recovery is an individual and subjective process, mental health care organizations can be recovery-oriented. The recovery of clients with SMI can be supported by, among other things, providing psychiatric rehabilitation services [11, 23].

Several rehabilitation methods have been developed to help people identify and achieve their own individual goals, including living independently, self-care, gaining and staying in employment, participating in routine educational settings, developing better relationships with their families, and pursuing leisure activities [24–27]. Comprehensive methods exist which focus on the personal goals and wishes of clients. Examples of well-known comprehensive rehabilitation methods are the Boston Psychiatric Rehabilitation (PR) approach [12] and the strengths model [28]. There are also rehabilitation methods which focus on a specific aspect of life, for example, ‘Individual Placement and Support’ (IPS) in which people are supported to gain and stay in competitive employment [29]. Finally, there are methods that aim at improving cognitive functioning or practical skills, e.g., cognitive remediation [30, 31] and cognitive adaptation training (CAT) [32, 33].

Internationally, there is an increasing amount of evidence for the effectiveness of the aforementioned interventions on social functioning [11, 13, 25, 26, 29, 34]. Swildens and colleagues [35] found that, among clients who participated in the Boston PR approach, goal attainment and social functioning were significantly higher compared with clients in the control condition. Furthermore, IPS has a strong effect on vocational outcomes [29, 36, 37]. The strengths model is associated with positive results on different outcomes [38–40] including decreased hospitalization and improved quality of life and social functioning [39, 41]. Although research on rehabilitation methods thus shows promising results, their effectiveness remains largely unknown. For example, few randomized controlled trials (RCTs) have been conducted to research the strengths model [38, 42], and most of these studies had methodological limitations such as small sample sizes and inadequate randomization [38]. Furthermore, in most of the studies only the effects on social functioning and quality of life were studied. Effects on personal recovery, hope and empowerment were not investigated, although these are also seen as an important outcome in mental health care nowadays. Finally, little is known about the effectiveness of these rehabilitation-oriented practices for clients of sheltered and supported housing facilities [43].

In the Netherlands, a rehabilitation method that is well known and often applied in mental health care is the Comprehensive Approach to Rehabilitation (CARe) methodology. The overall goal of the CARe methodology is to support a client in his/her recovery and to improve his/her quality of life. The central principles of this approach are: realizing goals and wishes; handling vulnerability; and improving the quality of the client’s social environment [44, 45]. The methodology is strongly influenced by the concept of ‘personal recovery’ and by the strengths model [28]. The CARe methodology is used in several mental health care organizations and organizations for sheltered and supported housing. It is suitable for all clients who experience psychosocial problems, regardless of the severity of their impairments or the phase of their recovery process. With regard to the CARe methodology, in contrast with the Boston PR approach, no controlled effect studies have yet been carried out [46, 47]. In the Netherlands, people with SMI often receive care from both mental health care organizations and housing facilities. Central in the approach of housing facilities is the focus on rehabilitation of their clients, while mental health care organizations focus more on treatment [43]. Several of these housing facilities make use of the CARe Methodology; therefore we chose these as research sites for this study.

This is, to our knowledge, the first RCT on the effectiveness of a method that combines rehabilitation, recovery and strengths principles. Finally, the CARe methodology is distinct from other methods due to the fact that it can be used for even the most vulnerable clients and not only the motivated ones. Hence, the aim of this study is to investigate the effectiveness of the CARe methodology on recovery, social functioning, quality of life, hope, empowerment, self-efficacy beliefs and needs for care of people with SMI.

## Methods/design

In this article, we follow the Consolidated Standard of Reporting Trials (CONSORT) 2010 statement on extension of the standard to cluster trials [48].

### Study design

**Fig. 1:**
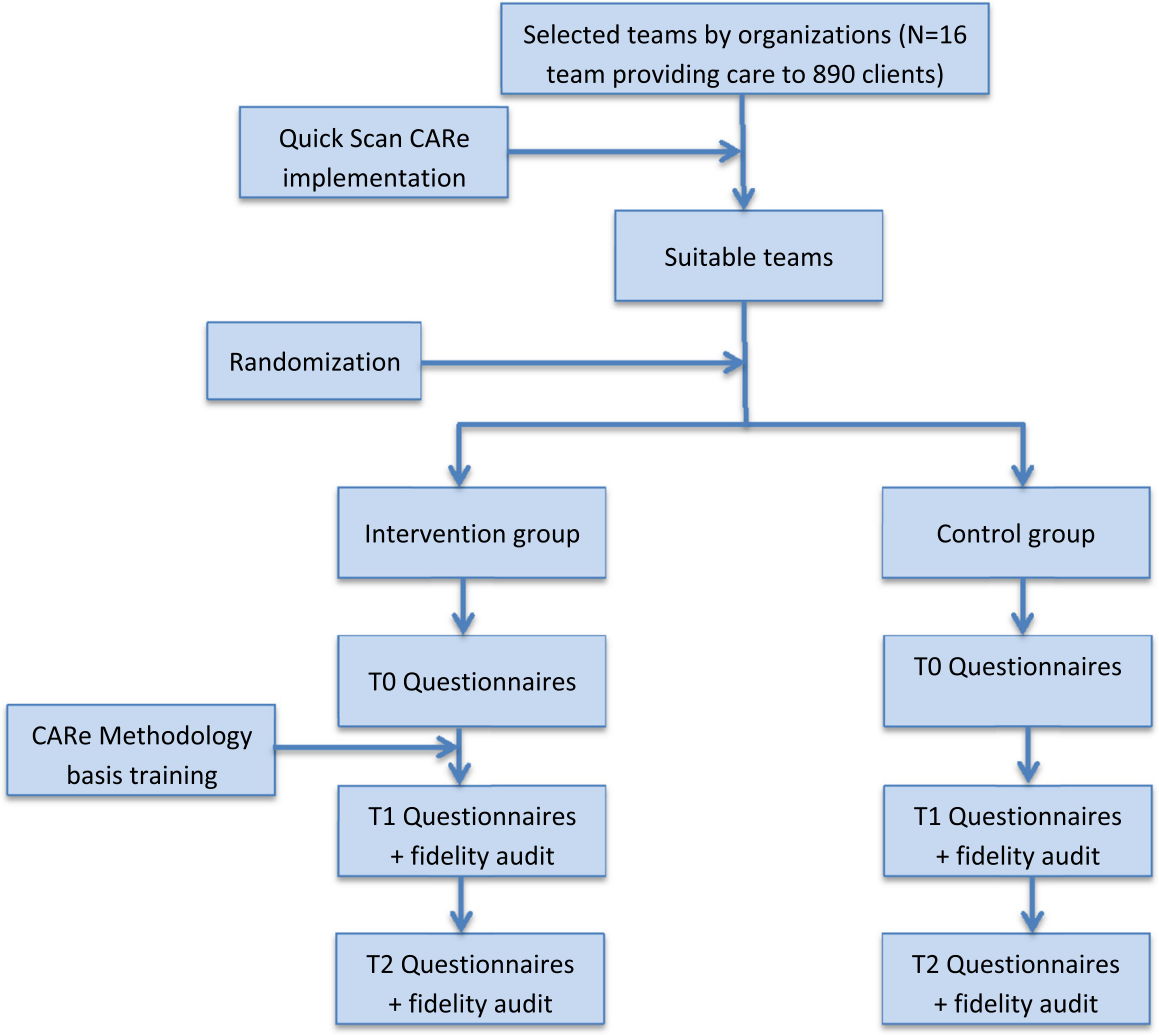
Flowchart of the study

This study is a two-armed cluster RCT that will be executed in teams selected from three organizations for sheltered housing in the Netherlands. These teams all provide sheltered housing and/or supported independent living services. Randomization will be applied at team level and will be stratified by organization. The professionals of the teams in the intervention group will receive a basic training in CARe methodology (three full-day meetings and four half-day meetings; see ‘Intervention’ for further information) while teams in the control group will continue to offer ‘care as usual’. Cluster randomization is necessary because the intervention is offered at team level; reorganization of this structure (i.e., reassigning clients to other teams in case of individual randomization) would disturb the clients’ living situations and relations of trust with their personal key workers, and would therefore be ethically undesirable. Furthermore, cluster randomization reduces contamination between the trial arms as much as possible. However, we will not be able to prevent staff changes completely; therefore we shall monitor this and take this into account in the analysis (see paragraphs outcome measures and statistical analysis). The participating teams will be randomized on an equal basis so an equal amount of teams and clients can participate in both arms. An independent researcher of the Department of Methodology and Statistics of Tilburg University will perform the randomization. The professionals and researchers will be aware of the allocation to the conditions; clients cannot be blinded but it will not be pointed out to them explicitly which condition they are in. All clients in the participating teams will be asked to participate in the study through an informed consent procedure. Standardized questionnaires will be completed at baseline (T0), and at 10 (T1) and 20 months (T2) post baseline (see Fig. 1). Besides client outcomes, the model fidelity of the CARe methodology will be assessed at T1 and T2.

The study has received ethical approval from the Medical Research Ethics Committee of the Elisabeth Hospital in Tilburg (NL41169.008.12). The trial registration number is ISRCTN77355880 (http://www.controlled-trials.com/ISRCTN77355880).

### Setting

The organizations for sheltered and supported housing in which this study is conducted provide ‘sheltered housing’ and ‘supported independent living’ services. In sheltered housing facilities, people with SMI live together and receive daily supervision from care professionals. In the case of supported independent living, the client lives independently, alone or with family or friends, and receives a certain amount of care at home. Both sheltered housing and supported independent living services are often provided by the same team of professionals. In that case the home base of the team is a sheltered housing facility, from where the team also provides supported independent living services to clients in the same area. The teams consist of ‘key workers’ and ‘support workers’. Key workers coordinate care around a client and draw up support plans and direct the execution of these plans. Support workers support clients in their daily living and are responsible for the execution of (parts of) the support plan. Furthermore, support workers take care of the living environment in a sheltered housing facility. Generally, key workers and support workers are educated as social workers or nurses. The teams are not responsible for the medical/psychiatric treatment of their clients. Most clients receive treatment from external (multidisciplinary) treatment teams from mental health care organizations.

### Interventions

#### CARe methodology

First the overall aims and corresponding theoretical background of the CARe methodology will be explained. Subsequently we will describe the way the methodology will be provided in practice.

#### Theoretical background

The central aim of the CARe methodology is improving the quality of life of people with a psychological or social vulnerability. The CARe methodology addresses this aim in three ways: (1) realizing the client’s wishes and goals; (2) handling vulnerability and reinforcing strengths; and (3) obtaining access to desired environments and improvement of the quality of the client’s living environment and social networks. The CARe methodology is strongly influenced by the concept of ‘personal recovery’ [17], the ‘presence approach’ [49] and the ‘strengths model’ [28, 44, 45, 50].

#### Personal recovery

One of the major objectives of the CARe methodology is to support clients in their personal recovery. In the CARe methodology, the recovery process consists of three phases: stabilization, reorientation and reintegration. When applying the CARe methodology, the individual recovery process of the client is central. In this respect, five clusters of recovery factors have to be investigated and reinforced. These clusters are: (1) motivation, (2) identity, (3) knowledge and skills, (4) social status and (5) social and material resources [44, 45, 50].

#### ‘Presence approach’

The ‘presence approach’ focuses on the professional’s attitude towards and relationship with the client. The fundamental idea of the presence approach is to create an equal relationship with the professional ‘being there’ for the client without focusing directly on the problems. Important attitudes in the presence approach are patience, unconditional attentiveness and receptivity [49]. When applying the CARe methodology, the presence approach is the central starting point of the way in which a worker builds a relationship with the client.

#### Strengths model

The third influence is the ‘strengths model’ of case management of Rapp [28]. The aim of the strengths model is to focus on the personal qualities, talents, and strengths of a person and his or her environment. The model has six principles: (1) focus on an individual’s strengths rather than pathology and limitations; (2) the case manager-client relationship is primary and essential; (3) interventions are based on clients’ wishes and choices; (4) the community is viewed as a source of possibilities, not as an obstacle; (5) the intervention is preferably offered in the community; and (6) people suffering from SMI can recover and continue to learn, grow and change. When working with the CARe methodology the worker and the client map the strengths of both the client and his/her environment, and use these strengths in achieving the clients goals [45, 50].

#### The CARe methodology in practice

**Fig. 2:**
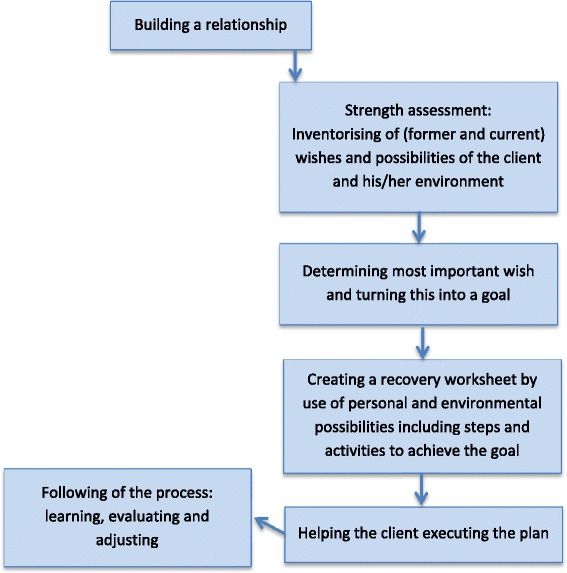
The CARe Methodology process

In practice, applying the CARe methodology consists of the following six steps (Fig. 2): (1) building and maintaining a constructive relationship with the client; (2) collecting information and making a ‘strengths assessment’ with the client. The strengths assessment can be used to gain an overview of a client’s former, current and desired situation in the fields of daily life, work, social contacts and leisure; (3) helping the client to formulate his/her wishes, make choices and set goals; (4) helping the client to complete a ‘recovery worksheet, this is a concrete plan with (small) tasks and activities that can be done to achieve the client’s goals and wishes’; (5) helping the client to execute the plan; and (6) after completing the process, to learn, evaluate and adjust [44, 45].

#### Training in the CARe methodology

The teams in the intervention group will receive basic training in the CARe methodology. The aims of this training are to train professionals in the principles of rehabilitative and recovery-supportive care and to support clients’ rehabilitation processes in a methodical way. The training consists of seven meetings, i.e., three full-day theory meetings and four half-day meetings in which the practical skills are learned. Qualified trainers from a specialized training institute conduct these meetings.

After the training program, the professionals continue to be supported in working according to the CARe methodology by means of CARe coaching meetings (once every 4–6 weeks) in which practical cases can be discussed. These coaching meetings are guided by a trained ‘CARe coach’ from the organization concerned, who is not a member of the workers’ teams.

#### Care as usual

The teams in the control group do not receive this training in CARe methodology. The workers in those teams will continue to work according to ‘care as usual’. Care as usual implies working according to the outdated CARe methodology and with a minimal level of model fidelity. Because the CARe Methodology is recently adapted, several distinctive differences exist between the outdated form of the methodology and the form the intervention teams will use. The most important difference between teams in the intervention group and teams in the control group teams will be that the control teams will not work with the ‘strengths assessment’ and the ‘recovery worksheet’, which are seen as the most important instruments of the current CARe Methodology. Besides that they will not be supported by the ‘CARe coaching meetings’. Finally, teams in the control group will be asked not to implement new practices oriented on recovery, rehabilitation or strengths for as long as they are participating in the study.

### Recruitment of teams

Because rehabilitation practices are common in sheltered and supported housing facilities in the Netherlands, it is impossible to include teams that do not work according to any rehabilitation method at all. However, to study the effects of the CARe methodology in a randomized design, teams are needed whose methodology is (1) outdated and (2) not adopted by the workers or inadequately applied. These teams will be selected in three steps. First, we will seek out sheltered housing organizations that possess an intention or interest in training their employees in the CARe methodology. Second, each such organization will be asked to make a selection of possible teams suitable for this study; teams in which (most of) the workers do not have training in CARe methodology, or were trained in an outdated version, and in which the CARe principles are downgraded due to, for example, changes of employees. Teams that are already trained in the current CARe methodology will be excluded from this study. Third, a researcher (NB) will interview the team leaders and make a definitive selection by means of the ‘Quick Scan CARe’, an instrument developed to map the general implementation of the CARe methodology in a team. Only teams with a very low level of implementation will be included in the study and randomly allocated to the intervention or the control group.

#### Team inclusion criteria

Teams of three organizations for sheltered and supported housing facilities in the Netherlands will be included. These teams provide sheltered housing and/or supported independent living services to adults with severe mental health problems. These teams work according to an outdated form of the CARe Methodology. Furthermore, in these teams the (outdated) CARe Methodology is not adopted by the workers or is inadequately applied.

### Recruitment of participants

All clients of the participating teams will be asked to participate in the study. An information meeting will be organized at the location and all clients will receive an information brochure. Subsequently clients will be approached individually by the researcher or via the staff.

The participating clients will be asked to give their informed consent in writing to take part in the data gathering and use of the data for the study. This informed consent will be signed before the start of the first interview. Each participant will be informed about his or her right to withdraw from the study at any time. Because the participating organizations already apply rehabilitation principles and specifically the CARe methodology is already part of the participating organizations no informed consent is needed for the group randomization and the receiving of care according to the CARe methodology.

#### Client inclusion and exclusion criteria

Adult clients (>18) who receive services from a team included in the study participate in the study. Clients with too little knowledge of the Dutch language to fill in the questionnaire and/or clients who are unable to give informed consent or participate in the study due to cognitive impairment or clinical symptoms will be excluded.

### Outcome measures

Outcome measures that suit the aims of the CARe methodology have been chosen. Furthermore, outcome measures have been selected on the basis of usage in comparable national and international research, and on their psychometric properties. Other considerations included were: expected effect sizes, sensitivity and interview duration. Based on these requirements the following outcomes and instruments are selected (see also Table 1):

**Table 1:** Outcomes and measure

Topic	Instrument	T0	T1	T2	Rater
Primary outcome measures (client level)					
Recovery	Mental Health Recovery Measure (MHRM)	x	x	x	Client
Societal functioning	Social Functioning Scale (SFS)	x	x	x	Client
Quality of life	Manchester Short Appraisal (MANSA)	x	x	x	Client
Secondary outcomes (client level)					
Empowerment	Dutch Empowerment Scale	x	x	x	Client
Hope	Herth Hope Index (HHI)	x	x	x	Client
Self-efficacy	Mental Health Confidence Scale (MHCS)	x	x	x	Client
Need for care	Camberwell Assessment of Needs (CANSAS)	x	x	x	Client
Additional process and control measures (client level)					
Demographic characteristics	Age, gender, nationality, level of education, marital status, living situation, principal daily pursuit, income	x	x	x	Client
Healthcare utilization	Diagnosis, psychiatric care, day care, contacts with care workers, (psychiatric) hospital admission, other care, psychiatric medication	x	x	x	Staff
Psychiatric symptoms	Brief Symptom Index (BSI)	x	x	x	Client
Recovery promoting relation	Recovery Promoting Relationship Scale (RPRS)	x	x	x	Client
Additional process and control measures (team level)					
Knowledge on recovery	Recovery Knowledge Inventory (RKI)	x	x	x	Staff
Fidelity of Care Methodology	CARe Methodology fidelity audit		x	x	Staff and clients
Quality of care	Quality Indicator for Rehabilitation Care (QUIRC)	x		x	Team leader

#### Primary outcomes

Because the CARe Methodology aims to support clients in their recovery and participation with the overall goal of increasing quality of life, we chose these three outcomes (recovery, social functioning and quality of life) as primary outcomes. All these outcomes will be measured by use of self-report measures.

- **Recovery** will be measured by the Dutch version of the Mental Health Recovery Measure (MHRM), an instrument developed to assess the recovery process of persons with SMI [19]. The MRHM is a self-report instrument with 30 items. The MHRM is a reliable and valid instrument. The instrument comprises three subscales: ‘self-empowerment’ (*α* = 0.90), ‘learning and new potentials’ (*α* = 0.86) and ‘spirituality’ (*α* = 0.94) [19]. All items are rated using a five-point Likert scale that ranges from ‘strongly disagree’ to ‘strongly agree’.
- The Social Functioning Scale (SFS) will be used to measure **social functioning**. The scale (*α* = 0.80) consists of 19 items and four checklists on seven domains: social engagement/withdrawal, interpersonal behaviour, pro-social activities, recreation, independence-competence, independence-performance and employment/occupation [51].
- **Quality of life** will be assessed by the Manchester Short Appraisal (MANSA), an instrument to measure quality of life in people with mental illness. The MANSA (*α* = 0.74) consists of 12 subjective items with a seven-point Likert scale (‘could not be worse’–‘could not be better’). Besides the subjective questions on satisfaction, the MANSA contains four yes/no questions, for example, about the presence of a good friend [52, 53].

#### Secondary outcomes

Besides the primary outcomes, secondary outcomes will be used, aiming to get more insight in the effects of the CARe Methodology. All these outcomes will be measured by use of self-report measures.

- **Empowerment** is the process of people achieving, or having the feeling that they have, control over their own lives. For the measurement of empowerment the Dutch Empowerment Scale (*α* = 0.93) will be used. This scale consists of 40 items distributed over six domains: professional help (*α* = 0.81), social support (*α* = 0.87), own wisdom (*α* = 0.89), belonging (*α* = 0.74), self-management (*α* = 0.74) and involvement in community (*α* = 0.81). The items are scored on a five-point Likert scale ranging from ‘strongly disagree’ to ‘strongly agree’ [54, 55].
- **Hope** will be assessed by the Dutch version of the Herth Hope Index (HHI), consisting of 12 four-point Likert scale items ranging from ‘strongly disagree’ to ‘strongly agree’. The Dutch version of the HHI consists of two factors, each of six items: ‘view on life and future’ (*α* = 0.8) and ‘self-confidence and inner strength’ (*α* = 0.69) (overall *α* = 0.84) [56, 57].
- The Dutch version of the Mental Health Confidence Scale (MHCS) will be used to measure health-related **self-efficacy beliefs** (*α* = 0.93). This scale has 16 items with a six-point Likert scale (‘totally no confidence’–‘full confidence’). The instrument has three subscales: optimism (six items, *α* = 0.87), coping (seven items, *α* = 0.76) and advocacy (three items, *α* = 0.93) [58, 59].
- **Need for care** will be measured by use of the 27-item client-rated version of the Camberwell Assessment of Needs Short Appraisal Schedule (CANSAS). With this instrument the client can score a health or social need as ‘no need’, ‘fulfilled need’ or ‘unfulfilled need’ [60].

#### Additional and control measures

In a complex research project such as this, there may be numerous external influences. Hence, several additional measures will be used to measure some factors that may modify or explain the possible effects.

- The following **demographic variables** will be measured: age, gender, marital status, nationality, educational status, employment status, income and living situation. These demographics will be measured by use of a client-rated form developed for the study.
- **Psychiatric symptoms** will be measured by use of the client-rated Brief Symptom Index (BSI) [61].
- The client-rated **Recovery Promoting Relationship Scale** (RPRS) (*α* = 0.90) will be used to measure to what extent the client experiences the relationship with his or her key worker as supporting his/her recovery. The scale consists of 24 items with a four-point Likert scale ranging from 1 (strongly disagree) to 4 (strongly agree) and with five indicating not applicable [62].
- **Worker’s knowledge of recovery** will be measured by use of the staff-rated Recovery Knowledge Inventory (RKI) (*α* = 0.80). The RKI consists of 20 items (scored on a five-point Likert scale ranging from strongly disagree to strongly degree) [62, 63]. Some additional questions will be added to the RKI concerning age, level and type of education and whether the worker received a CARe methodology training. All workers in the participating teams will be asked to fill in the RKI.
- The key workers of the participating clients will be asked to answer questions regarding the psychiatric **diagnosis** (DSM IV) of the client and the **amount of contact** they have with the client (hours per day and/or week). Besides that*,* there will be questions about the client’s **care consumption** in general and his or her use of work/recreation facilities (hours per week).

#### Model fidelity of the CARe methodology

At T1 and T2, a ‘CARe methodology fidelity audit’ will be performed for all the teams aiming to investigate the extent to which the teams work according to the critical ingredients of the CARe methodology. These critical ingredients are: (1) the presence approach, (2) recovery orientation, (3) strengths orientation, (4) working according to the steps of the CARe methodology, (5) each client has a key worker, and (6) certification, learning (coaching) and implementation. This audit consists of individual interviews with clients, key and support workers, the team leader or manager and a CARe coach. These interviews will be conducted by two auditors: a researcher (NB) and an independent CARe coach. This audit will result in a report with quantitative scores on each critical ingredient of the CARe methodology and a total score. This audit will be performed for teams in the intervention group as well as the teams in the control group so that differences in the model fidelity levels between the two groups can be detected. The results of the audits will be used to investigate to what extent client outcomes can be related to the level of implementation of the CARe methodology and its critical ingredients.

#### Quality of care

To assess the overall quality of care at the team level, the Quality Indicator for Rehabilitation Care (QuIRC) is used. The QuIRC is a European instrument developed to assess quality of care delivered in hospitals and community-based mental health units [64]. The QuIRC comprises 145 questions on service quality and provision (e.g., number of beds, treatments and interventions, training and supervision of staff). The QuIRC provides ratings across seven areas of care: built environment, therapeutic environment, treatment and interventions, self-management and autonomy, social interface; human rights and recovery oriented care [64]. In this way we can investigate to what extent the implementation of the CARe methodology influences the overall quality of care; and relate the areas of care to the outcomes of the other instruments on client level as well as on team level. The QuIRC will be filled in by a researcher (NB) based on face-to-face interviews with the team managers.

### Power calculation/sample size

Sample size was calculated taking into account the design effect (due to group randomization) and the expected effect size. The measures with the highest expected effect size within the duration of the study of 20 months are: empowerment (0.38) and hope (0.50) [26, 43]. Cohen’s d was used as the measure for effect size with *α* = 0.05 and a power of 0.80, based on a two-sided test. The design effect used is estimated to be 1.5 based on an average cluster size of 38 and an intra-cluster correlation (ICC) of 0.013. Based on the effect size of empowerment (0.38; the lowest of the two above mentioned) a sample of 128 clients per condition is needed. When taking into account a reduction of 20 % for loss due to follow up, 160 clients per group will be recruited to achieve the required power. To reach a sufficient amount of clients 16 teams will be included in the study, which together provide services to 890 clients.

### Statistical analysis

Data will be analysed according to intention-to-treat, meaning that participants will be analysed in the group to which they were allocated by randomization [65]. SPSS 19 will be used for the analysis. Because the study has a cluster randomized design, longitudinal multilevel analysis (linear mixed modeling with random intercepts at both team level and individual level) is the analysis method of choice. First, effectiveness of the CARe Methodology on the three primary outcomes, recovery, social functioning and quality of life, will be evaluated. Subsequently, the effectiveness on four secondary outcomes, hope, empowerment, self-efficacy beliefs and need for care will be evaluated. An alpha correction (i.e., Bonferroni adjustment) will be applied across analysis of the primary measures in order to maintain a family-wise alpha level of 0.05. A separate Bonferroni adjustment will be applied to the set of analysis for the secondary measures to maintain their family-wise alpha level at 0.05. Furthermore, in separate analyses we will assess whether different types of predictors explain the outcomes: (1) client characteristics (age, gender, having a partner, type of housing, diagnosis), (2) symptom severity (BSI), (3) health and day care utilization. Only predictors that influence the prediction of the outcome measures will be added tot the final model. Outcomes will be measured at 10 months and 20 months post-baseline (time will be analyzed as a categorical variable).

To detect significant differences in the baseline characteristics between the intervention group and control group descriptive analysis will be used. When necessary these differences will be taken into account in the analysis. Missing data and drop-outs will be analysed and accounted for by multiple imputation if the assumption of data missing at random (MAR) is not violated [66].

## Discussion

This article describes the design of a cluster-randomized controlled trial which aims to investigate the effectiveness of the CARe methodology on (among other things) quality of life, social participation and recovery. This study is the first effect study on the CARe methodology and one of the few studies with a control group on a comprehensive rehabilitation method or strengths based approach [25, 38]. This study is of high relevance because recovery and rehabilitation oriented care has become increasing important for mental health care organizations, especially nowadays as de-institutionalization and participation in society is increasingly being encouraged [14, 43, 67].

The strength of this study is that a broad group of clients with long-term SMI (elderly, double diagnosis, mild intellectual disabilities, inpatient and outpatient) will be included. Most rehabilitation or recovery-oriented interventions are offered only to clients who are motivated to participate in them [35, 55]. Consequently, research on these interventions tends to include only motivated clients. The CARe methodology is for all persons with SMI, and therefore this study includes all clients who choose to participate in the interviews for the study, regardless of their rehabilitation readiness or phase of recovery. The underlying reason for this is that the CARe methodology is a method developed for all kinds of clients, including vulnerable ones. Due to this broad inclusion the participants in this study can be seen as representative of clients with (long-term) SMI. This is not only interesting for the analysis of the effects of the CARe methodology, but it also gives insight into where this group stands in terms of societal participation, recovery, hope, quality of life, and empowerment. Therefore the results of this study will add to our current knowledge.

Another strength of this study is that it includes assessment of the level of implementation of the CARe methodology. In this ‘fidelity audit’ interviews will be conducted with clients, workers, team leaders and CARe coaches on different aspects of CARe methodology. This will make it possible to attribute the outcomes to the level of implementation and/or to specific elements of it. Moreover it will give insight into the most critical elements of the method. This audit is at the same time a limitation because the instrument is not yet fully investigated and validated.

Another limitation of the study is that the effects of the CARe training may be biased because several principles of rehabilitation and recovery are already used in regular practice to some extent, which might bias the ‘care as usual’ condition. However, with the selection procedure designed for the participating teams (pre-selection by the organizations, quick scan) and the fidelity audits in both conditions, we prevent and control for this as much as possible. Also, the fact that professionals as well as clients cannot be blinded for the intervention is a limitation of this study design. It is generally known that it is very difficult to investigate the effectiveness of a complex social intervention in a practical environment in which several influences play a role [68]. Nevertheless, in this study these influences can be taken into account, because they will be measured on individual, organizational and environmental levels. Hence the effects of the CARe methodology can be studied in the complex context of practice.

This study will provide insight into the recovery processes of people with SMI and the effects of a comprehensive rehabilitation method on these processes. The results can be used to improve the CARe methodology and the corresponding training program. Furthermore, the results can contribute to the development of recovery-oriented care in general and the inclusion of people with SMI.
